# Factors influencing the outcomes of Community Treatment Orders: state-wide study using linked administrative health data from New South Wales, Australia

**DOI:** 10.1192/bjo.2026.10987

**Published:** 2026-03-10

**Authors:** Claudia Bull, Grant Sara, Christopher James Ryan, Lisa Brophy, Tessa Zirnsak, Giles Newton-Howes, Chris Maylea, Edwina Light, Sharon Lawn, Penelope Weller, Vrinda Edan, Steve Kisely

**Affiliations:** Queensland Centre for Mental Health Research, Faculty of Health, Medicine and Behavioural Sciences, The University of Queensland, Brisbane, Queensland, Australia; Princess Alexandra Hospital Southside Clinical Unit, Faculty of Health, Medicine and Behavioural Sciences, The University of Queensland, Brisbane, Queensland, Australia; The ALIVE National Centre for Mental Health Research Translation, Faculty of Health, Medicine and Behavioural Sciences, The University of Queensland, Brisbane, Queensland, Australia; InforMH, System Information and Analytics Branch, NSW Ministry of Health, Sydney, New South Wales, Australia; Northern Clinical School, Faculty of Medicine and Health, University of Sydney, Sydney, New South Wales, Australia; Faculty of Medicine and Health, University of New South Wales, Sydney, New South Wales, Australia; Discipline of Psychiatry and Mental Health, University of New South Wales, Sydney, New South Wales, Australia; School of Medicine, University of Notre Dame Sydney, Sydney, New South Wales, Australia; Department of Psychiatry, St Vincent’s Hospital, Darlinghurst, New South Wales, Australia; The ALIVE National Centre for Mental Health Research Translation, La Trobe University, Melbourne, Victoria, Australia; Social Work and Social Policy, Department of Community and Clinical Health, School of Allied Health, Human Services and Sport, La Trobe University, Melbourne, Victoria, Australia; Department of Psychological Medicine, University of Otago, Wellington, New Zealand; School of Law, La Trobe University, Melbourne, Victoria, Australia; School of Public Health, Faculty of Medicine and Health, The University of Sydney, Sydney, New South Wales, Australia; Lived Experience Australia, Adelaide, South Australia, Australia; College of Medicine and Public Health, Flinders University, Adelaide, South Australia, Australia; School of Law, RMIT University, Melbourne, Victoria, Australia; Consumer Academic Program, Centre for Mental Health Nursing, Medicine, Dentistry and Health Sciences, The University of Melbourne, Melbourne, Victoria, Australia; Metro South Addiction and Mental Health Service, Brisbane, Queensland, Australia; Departments of Psychiatry, Community Health and Epidemiology, Dalhousie University, Halifax, Nova Scotia, Canada

**Keywords:** Community Treatment Orders, involuntary, out-patient commitment, administrative health data

## Abstract

**Background:**

The effectiveness of Community Treatment Orders (CTO) and the variability with which they are used remains the subject of ongoing debate.

**Aims:**

To examine the associations between discharge from psychiatric in-patient care on to a CTO in New South Wales (NSW), Australia, and hospital admissions and bed-days in the following 12 months.

**Method:**

Retrospective matched case-control study using linked administrative health data from NSW between 1 January 2017 and 31 December 2023. Cases were individuals discharged on to a CTO after their first psychiatric hospital admission during the study period. We attempted to match controls 2:1 on age, gender and hospital discharge within 6 months of each other. Data were from the NSW Mental Health Ambulatory and Admitted Patient Data Collections.

**Results:**

There were 5506 individuals discharged on to CTOs and 9761 matched controls. Discharge on to a CTO did not affect the odds of hospital readmissions in the following 12 months (adjusted odds ratio (OR_adj_) = 1.06, 95% CI 0.97–1.14) though was associated with significantly greater bed-days (log *β*_adj_ = 0.12, 95% CI 0.08–0.17, *p* < 0.0001). Individuals with a principal diagnosis of non-affective psychosis who were discharged on to a CTO had significantly lower odds of hospital readmissions in the following 12 months (OR_adj_ = 0.67, 95% CI 0.59–0.77).

**Conclusions:**

Discharge on to a CTO did not significantly affect hospital readmissions across the full sample, but did significantly lower the odds for individuals with non-affective psychosis. This suggests that targeted use of CTOs in specific populations (e.g., non-affective psychosis) warrants greater consideration, as the benefit of their use otherwise – especially from a human rights point of view – is unclear.

**Registration:**

Australian and New Zealand Clinical Trials Registry (ACTRN12624000152527).

Community Treatment Orders (CTOs, also referred to as compulsory community treatment) are legal orders that require some individuals with severe mental illnesses (SMI) to accept community/out-patient mental healthcare. Someone placed on a CTO must have regular contact with mental health services and accept medication when prescribed while living in the community.^1^ CTOs differ from in-patient treatment orders which mandate compulsory treatment in an in-patient facility when no less restrictive treatment options are reasonably available to protect patients themselves or others from serious harm.^2^ CTOs enable mandated community-based treatment with the option for treating teams to recall individuals to hospital if required.^3^

The effectiveness of CTOs remains a subject of ongoing debate. While some observational studies, particularly those using before-and-after designs, suggest that CTOs may reduce hospital readmissions, in-patient bed-days and improve treatment adherence in the community, these findings are often tempered by methodological limitations including regression to the mean and a lack of control group.^4,5^ In contrast, controlled studies, including randomised controlled trials (RCTs), have generally reported limited or non-significant effects on outcomes such as hospital readmissions, treatment engagement and psychosocial functioning.^4,6,7^ Notably, some observational evidence indicates that potential benefits may be more apparent with longer CTO durations or in jurisdictions where CTOs are applied more judiciously, suggesting that context and targeted use may influence their effectiveness.^5,6^

The variability with which CTOs are used also remains a topic of considerable debate. Australia has some of the highest rates of CTO use internationally although there is substantial variation within and between each of Australia’s eight states and territories.^3,8^ For instance, in a study of four Australian jurisdictions, CTO use as a proportion of people in contact with mental health services ranged from 8.0 to 17.6%, with New South Wales reporting the lowest rates and South Australia the highest.^9^ While legislative differences on CTO eligibility across jurisdictions likely plays a role, this variation raises important ethical and law reform questions, particularly as rates of SMI do not vary as considerably.^10^

There is growing interest in identifying patient subgroups who may benefit more from CTOs. A recent case-control study in Queensland found that individuals with non-affective psychosis had significantly lower odds of readmission and fewer bed-days in the 12 months following CTO discharge, even after adjusting for key covariates.^11^ Similarly, a New Zealand on–off study showed that people with non-affective psychosis experienced fewer admissions and bed-days while on a CTO compared to periods of voluntary care, whereas those with other diagnoses showed the opposite trend.^12^ Although the New Zealand study lacked a non-CTO control group, both studies suggest differential outcomes by diagnosis. Supporting this, a meta-regression of Australian studies found greater reductions in in-patient service use in jurisdictions where CTOs were used more selectively for individuals with non-affective psychosis.^6^

The aim of this study was therefore to investigate the impact of discharge from psychiatric in-patient care in New South Wales (NSW) on to a CTO, on hospital admissions and bed-days in the following 12 months. Secondary to this, we sought to understand the sociodemographic and health service use characteristics associated with being discharged on to a CTO in NSW, as well as whether hospital admissions and bed-days in the subsequent 12 months differed relative to an individual’s principal diagnosis. We specifically focused on NSW as it is the most populous state in Australia (>8 million residents) with one of the lowest rates of CTO use, more closely aligning with international rates.^13^

## Method

### Study design

This retrospective case-control study is part of the Factors Affecting Community Treatment Orders Research Study (FACTORS).^14^ FACTORS is a multi-methods programme of research examining the disproportionate impact of CTOs on certain subgroups of the Australian population, and the reasons for wide variation in their use. For the purposes of this study, we used state-wide NSW linked administrative health data from 1 January 2017 to 31 December 2023.

The authors assert that all procedures contributing to this work comply with the ethical standards of the relevant national and institutional committees on human experimentation and with the Helsinki Declaration of 1975, as revised in 2013. We received ethical approval from the Metro South Human Research Ethics Committee (HREC/2023/QMS/94340), granting us a waiver of consent. The study was retrospectively registered as an observation study with the Australian and New Zealand Clinical Trials Registry (ACTRN12624000152527). We followed the STrengthening the Reporting of OBservational studies in Epidemiology (STROBE) guidelines.^15^

### Participants

Cases were people aged 18 years or older and discharged on to a CTO after a psychiatric hospital admission between 1 January 2018 and 31 December 2022. The first (index) psychiatric admission per person during the observation period was examined. Historical data from 1 January 2017 captured psychiatric and non-psychiatric admissions and community mental health contacts in the 12 months preceding the index admission. Participants were followed for up to 12 months post-discharge (through to 31 December 2023).

CTO status was identified using legal status code 3A (ambulatory or in-patient CTO) in the NSW Mental Health Ambulatory Data Collection (MH-AMB). We considered someone to be ‘discharged on to a CTO’ if their legal status was 3A during the index psychiatric admission or within 14 days of discharge. Controls were people discharged into voluntary community mental healthcare, who accessed community mental healthcare services within 30 days of the index date to be considered eligible as this was indicative of active voluntary mental health treatment. Over 95% of potential control group members accessed community mental health services within 30 days of discharge. We attempted to match controls to cases 2:1 on age, gender and hospital discharge within 6 months of each other, though ended up with a matching ratio of 1.8:1. Supplementary file 1 (available at https://doi.org/10.1192/bjo.2026.10987) illustrates an example of a case-control match.

### Data sources and variables

In Australia, mental health services are provided by public and private institutions under federal and state government, insurer and individual funding arrangements.^16^ We linked NSW MH-AMB and NSW Admitted Patient Data Collection (APDC). NSW MH-AMB comprises activity-based data on the assessment, treatment and rehabilitation care of non-admitted patients including psychiatric out-patient services, mental health day programmes and outreach services in publicly provided facilities.^17^ One person could have multiple activity records across multiple service contacts in the NSW MH-AMB. NSW APDC comprises episodes of care-based data on all admitted patient services provided by public hospitals, private hospitals, psychiatric hospitals, multi-purpose public services and private day procedure centres.^17^ One person could have multiple episodes of care recorded in the NSW APDC. Individuals were eligible for inclusion in the study if the date of their first (index) discharge from psychiatric hospital admission on to a CTO or voluntary community mental healthcare was between 1 January 2018 and 31 December 2022. This could have been someone’s first ever discharge on to a CTO or one of many discharges on to a CTO, but the first to fall within the study period. This date represented the baseline for follow-up for all subsequent analyses.

The outcomes of interest in this study were hospitalisations and median bed-days in the 12 months after the index psychiatric admission. We examined all hospital readmissions and bed-days, as well as psychiatric-specific readmissions and bed-days, in recognition that this population often experiences complex comorbidities that may lead to both psychiatric and non-psychiatric admissions. While these are imperfect indicators of potential CTO benefit in that they fail to consider the lived experience of someone on a CTO, other psychosocial outcomes or treatment and medication adherence, they are the best available indicators in administrative health data collections. Moreover, they are widely used in the literature,^6,7^ enabling comparison across studies, and are linked to key clinical and legislative justifications for CTO use.

To determine the sociodemographic and health service use characteristics associated with these outcomes, we included age (≤40 years old versus >40 years old), marital status (unpartnered/never married versus partnered/married), sex (male versus female), rurality of local health district (LHD, metropolitan area versus remote/rural area), country of birth (Australia, New Zealand and north-west Europe versus elsewhere), preferred language (English versus all others), principal diagnosis (non-affective psychosis including schizophrenia and drug-induced psychosis, mood disorders and all other disorders), psychiatric admissions in the 12 months prior to the index date (any versus none), non-psychiatric admissions in the 12 months prior to the index date (any versus none) and community mental health service use in the 12 month prior to the index date (less than or equal to the median number of community contacts versus greater than the median number of community contacts). These characteristics have previously shown to be associated with CTO placement.^11,13,18^

Principal diagnosis was based on the primary ICD-10-AM code listed for the index psychiatric admission. ICD-10-AM codes were grouped accordingly: non-affective psychosis (otherwise described as schizophrenia spectrum disorder) including schizophrenia and drug-induced psychosis (F1x.5, F1x.7, F20–29); mood disorders (F30–39); and all other disorders (all other F codes). The ‘all other disorders’ group included remaining ICD-10-AM F codes aggregated to ensure adequate cell sizes for analysis. We grouped country of birth into Australia, New Zealand and north-west Europe vs elsewhere because north-west Europe (defined according to the ABS Standard Australian Classification of Countries (SACC)^19^) represented the smallest permissible regional unit available in our dataset to capture individuals born in the United Kingdom (UK) and Ireland. These countries are comparable to Australia and New Zealand in terms of CTO legislation, mental health system structure and service-use patterns. In line with prior research, this grouping was used to ensure accurate analysis of country of birth-related disparities in CTO placement^11,20^ Additionally, we examined whether hospitalisations and median bed-days in the 12 months after the index psychiatric admission differed depending on the principal diagnosis, as well median community mental health appointments in the 12 months after the index psychiatric admission.

### Statistical analysis

After assessing data normality, we described sociodemographic and health service use characteristics using means, standard deviations, frequencies, proportions, medians and interquartile ranges (IQR). We examined associations between sociodemographic and health service use characteristics (independent variables) and being discharged on to a CTO after the index psychiatric admission (dependent variable), as well as hospitalisations in the 12 months after the index psychiatric admission (dependent variable), using bivariate and multivariate logistic regression models. Bivariate models assessed independent associations, while multivariate models included all variables as covariates. Principal diagnosis was dichotomised (e.g., non-affective psychosis including schizophrenia and drug-induced psychosis versus all other diagnoses) in these regression models. Sensitivity analyses were also conducted to examine the associations between sociodemographic and health service use characteristics (independent variables) and psychiatric-specific hospitalisations in the 12 months after the index admission (dependent variable).

We also examined associations between sociodemographic and health service use characteristics (independent variables) and bed-days in the 12 months after the index psychiatric admission (dependent variable) using bivariate and multivariate linear regression models. Due to right-skewed bed-days, a constant of one was added to all values and data were logarithmically transformed.^21^ Sensitivity analyses were also conducted to examine the associations between sociodemographic and health service use characteristics (independent variables) and psychiatric-specific bed-days in the 12 months after the index admission (dependent variable).

Finally, within the subgroup of individuals discharged on to a CTO, we examined whether hospitalisations and median bed-days in the 12 months after the index psychiatric admission differed according to principal diagnosis. For these analyses, each diagnostic category was compared against all other diagnostic groups combined (e.g., individuals with non-affective psychosis were compared to all others without this diagnosis). Logistic regression was used for hospitalisations in the 12 months after the index psychiatric admission, and linear regression for log-transformed bed-days. A sensitivity analysis further examined non-affective psychosis excluding drug-induced psychosis (retaining ICD-10-AM codes: F20–29) using the same comparison framework. Additional sensitivity analyses examined whether psychiatric-specific hospitalisations and median bed-days in the 12 months after the index admission differed according to principal diagnosis. Using the Wilcoxon Rank-Sum test, we also compared the median community mental health appointments in the 12 months after the index psychiatric admission across principal diagnosis groups. Significance was set at *p* ≤ 0.05, and all analyses were performed in SAS 9.4 (SAS Institute Inc, North Carolina, USA; https://www.sas.com/en_us/home.html) via the Secure Unified Research Environment workspace (Sax Institute, New South Wales, Australia; https://www.saxinstitute.org.au/solutions/sure/).

We did not undertake causal inference analyses because several of the key assumptions required for valid counterfactual estimation could not be satisfied. Specifically, essential confounders that influence placement on CTOs and subsequent service use (e.g., illness severity, patient insight, social support, treatment adherence and clinical risk assessment) were not available in the administrative data.

## Results

The sample consisted of 5506 people (36.1%) discharged on to a CTO and 9761 controls (63.9%) who entered voluntary community mental healthcare after the index psychiatric admission (sample total *n* = 15 267). The sociodemographic and health service use characteristics of both groups are described in Supplementary file 2. Table 1 shows the sociodemographic and health service use characteristics associated with being discharged on to a CTO after the index psychiatric admission. After adjustment for all other variables in the table, people were more likely to be discharged on to a CTO if they were unpartnered/ had never been married (adjusted odds ratio (OR_adj_) = 1.59, 95% CI 1.46–1.73, *p* < 0.0001), were born outside of Australia, New Zealand and north-west Europe (OR_adj_ = 1.35, 95% CI 1.20–1.51, *p* < 0.0001), had a principal diagnosis of non-affective psychosis including schizophrenia and drug-induced psychosis (OR_adj_ = 5.05, 95% CI 4.66–5.47, *p* < 0.0001), had any psychiatric admission in the previous 12 months (OR_adj_ = 1.17, 95% CI 1.06–1.30, *p* = 0.002) or had greater than the median number of community mental health service contacts in the previous 12 months (OR_adj_ = 3.53, 95% CI 3.25–3.83, *p* < 0.0001). They were less likely to be discharged on to a CTO if they were male (OR_adj_ = 0.79, 95% CI 0.73–0.86, *p* < 0.0001).

**Table 1:** Sociodemographic and health service use characteristics associated with being discharged on to a CTO at or following the index psychiatric admission

Characteristic	Voluntary group 9761 (63.9%) *n* (%)	CTO group 5506 (36.1%) *n* (%)	OR (95% CI)	OR_adj_ (95% CI)^a^	Sig.
Age: ≤40 years old	4519 (46.3)	2549 (46.3)	1.00 (0.94–1.07)	0.95 (0.88–1.03)	0.228
Relationship status: Unpartnered/never married	4848 (49.7)	3734 (67.8)	2.14 (1.99–2.29)	1.59 (1.46–1.73)	**<0.0001**
Sex: Male	5904 (60.5)	3353 (60.9)	1.02 (0.95–1.09)	0.79 (0.73–0.86)	**<0.0001**
Rurality of residence: Metropolitan area	5455 (55.9)	3353 (60.9)	1.23 (1.15–1.31)	1.05 (0.97–1.14)	0.233
Country of birth: Outside of Australia, New Zealand and north-west Europe^b^	1602 (16.4)	1150 (20.9)	1.34 (1.24–1.46)	1.35 (1.20–1.51)	**<0.0001**
Preferred language: Other than English	601 (6.2)	424 (7.7)	1.27 (1.12–1.45)	0.97 (0.82–1.15)	0.743
Principal diagnosis: Non-affective psychosis (incl. schizophrenia)	2945 (30.2)	4108 (74.6)	6.80 (6.31–7.33)	5.05 (4.66–5.47)	**<0.0001**
Any psychiatric admissions in previous 12 months^c^	2144 (22.0)	1882 (34.2)	1.84 (1.71–1.99)	1.17 (1.06–1.30)	**0.002**
Any non-psychiatric admissions in previous 12 months^c^	3611 (37.0)	1688 (30.7)	0.75 (0.70–0.81)	0.95 (0.86–1.04)	0.245
Any community mental health appointments in previous 12 months, split by median	3532 (36.2)	4067 (73.9)	4.98 (4.63–5.36)	3.53 (3.25–3.83)	**<0.0001**

CTO, Community Treatment Order; OR, odds ratio; OR_adj_, adjusted odds ratio; Sig., significance; incl., including.

a. Adjusted for all variables in this table.

b. North-west Europe includes: UK, Channel Islands and Isle of Man (including England, Isle of Man, Northern Ireland, Scotland, Wales, Guernsey and Jersey), Ireland, western Europe (including Austria, Belgium, France, Germany, Liechtenstein, Luxembourg, Monaco, The Netherlands and Switzerland), northern Europe (including Denmark, Faroe Islands, Finland, Greenland, Iceland and Aland Islands).

c. Excluding the index psychiatric admission.

Bold results indicate statistical significance (*p* < 0.05).

Approximately 41% of the sample were admitted to hospital in the 12 months following their index psychiatric admission. Table 2 shows that people were significantly less likely to be admitted in the following 12 months if they were 40 years old or younger (OR_adj_ = 0.88, 95% CI 0.82–0.94, *p* < 0.001), born outside of Australia, New Zealand or north-west Europe (OR_adj_ = 0.70, 95% CI 0.63–0.78, *p* < 0.0001), had a preferred language other than English (OR_adj_ = 0.79, 95% CI 0.67–0.92, *p* = 0.003) or had a principal diagnosis of non-affective psychosis including schizophrenia and drug-induced psychosis (OR_adj_ = 0.72, 95% CI 0.66–0.77, *p* < 0.0001). They were significantly more likely to be admitted if they lived in a metropolitan area (OR_adj_ = 1.21, 95% CI 1.12–1.30, *p* < 0.0001), had any psychiatric admissions in the previous 12 months (OR_adj_ = 2.83, 95% CI 2.59–3.09, *p* < 0.0001), had any non-psychiatric admissions in the previous 12 months (OR_adj_ = 2.12, 95% CI 1.95–2.29, *p* < 0.0001) and had greater than the median number of community mental health service contacts in the previous 12 months (OR_adj_ = 1.35, 95% CI 1.26–1.46, *p* < 0.0001). Discharge on to a CTO after the index psychiatric admission had no significant association with admissions in the subsequent 12 months (OR_adj_ = 1.06, 95% CI 0.97–1.14, *p* = 0.190). Results were similar in sensitivity analyses restricted to psychiatric-specific hospital admissions in the following 12 months (Supplementary file 3), though discharge on to a CTO after the index admission was significantly associated with psychiatric-specific hospital admissions in the following 12 months (OR_adj_ = 1.36, 95% CI 1.24–1.49, *p* < 0.0001).

**Table 2:** Sociodemographic and health service use characteristics associated with any hospital admissions in the 12 months after the index psychiatric admission

Characteristic	Not admitted 8992 (58.9%) *n* (%)	Admitted 6275 (41.1%) *n* (%)	OR (95% CI)	OR_adj_ (95% CI)^a^	Sig.
Age: ≤40 years old	4281 (47.7)	2781 (44.3)	0.87 (0.82–0.93)	0.88 (0.82–0.94)	**<0.001**
Relationship status: Unpartnered/never married	5097 (56.7)	3484 (55.5)	0.95 (0.89–1.02)	0.97 (0.90–1.04)	0.418
Sex: Male	5555 (61.8)	3702 (59.0)	0.89 (0.83–0.95)	0.97 (0.90–1.04)	0.335
Rurality of residence: Metropolitan area	5059 (56.3)	3748 (59.7)	1.15 (1.08–1.23)	1.21 (1.12–1.30)	**<0.0001**
Country of birth: Outside of Australia, New Zealand and north-west Europe^b^	1824 (20.3)	928 (14.8)	0.68 (0.62–0.74)	0.70 (0.63–0.78)	**<0.0001**
Preferred language: Other than English	699 (7.8)	326 (5.2)	0.65 (0.57–0.74)	0.79 (0.67–0.92)	**0.003**
Principal diagnosis: Non-affective psychosis (incl. schizophrenia)	4361 (48.5)	2692 (42.9)	0.80 (0.75–0.85)	0.72 (0.66–0.77)	**<0.0001**
Any psychiatric admissions in previous 12 months^c^	1834 (20.4)	2192 (34.9)	2.09 (1.95–2.25)	2.83 (2.59–3.09)	**<0.0001**
Any non-psychiatric admissions in previous 12 months^c^	2843 (31.6)	2456 (39.1)	1.39 (1.30–1.49)	2.12 (1.95–2.29)	**<0.0001**
Any community mental health appointments in previous 12 months, split by median	4066 (45.2)	3533 (56.3)	1.56 (1.46–1.67)	1.35 (1.26–1.46)	**<0.0001**
Discharged on to a CTO after index hospital admission	3176 (35.3)	2330 (37.1)	1.08 (1.01–1.16)	1.06 (0.97–1.14)	0.190

CTO, Community Treatment Order; OR, odds ratio; OR_adj_, adjusted odds ratio; Sig., significance; incl., including.

a. Adjusted for all variables in this table.

b. North-west Europe includes: UK, Channel Islands and Isle of Man (including England, Isle of Man, Northern Ireland, Scotland, Wales, Guernsey and Jersey), Ireland, western Europe (including Austria, Belgium, France, Germany, Liechtenstein, Luxembourg, Monaco, The Netherlands and Switzerland), northern Europe (including Denmark, Faroe Islands, Finland, Greenland, Iceland and Aland Islands).

c. Excluding the index admission.

Bold results indicate statistical significance (*p* < 0.05).

**Fig. 1:**
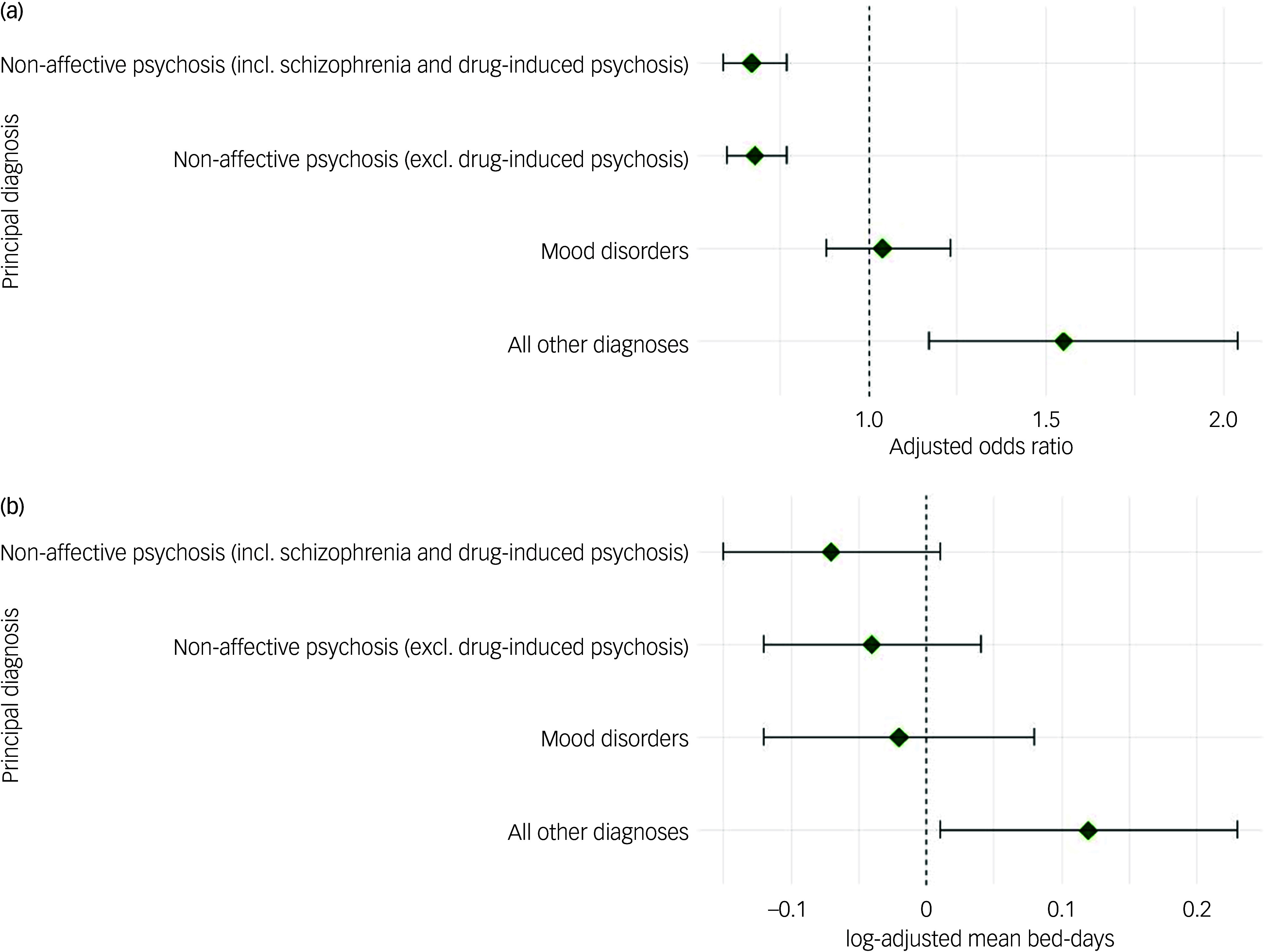
(a) Adjusted odds of hospital admissions in the 12 months after index psychiatric admission for people discharged on to a Community Treatment Orders (CTO), stratified by principal diagnosis. (b) log-adjusted bed-days in the 12 months after index psychiatric admission for people discharged on to a CTO, stratified by principal diagnosis. Incl., including; excl., excluding.

Of those in the CTO group, people who had a principal diagnosis of non-affective psychosis including schizophrenia and drug-induced psychosis had significantly lower odds of being admitted to hospital in the following 12 months (OR_adj_ = 0.67, 95% CI 0.59–0.77, *p* < 0.0001). Sensitivity analysis excluding people with drug-induced psychosis revealed slightly lower odds of being admitted to hospital in the following 12 months (OR_adj_ = 0.68, 95% CI 0.60–0.77, *p* < 0.0001). Mood disorders were not associated with increased odds of hospital admissions, though all other diagnoses were (Table 3 and Fig. 1(a)). Again, similar results were seen in sensitivity analyses restricted to psychiatric-specific hospital admissions in the following 12 months (Supplementary file 3).

**Table 3:** Odds of hospital readmissions over 12 months of follow-up for people discharged on to CTOs after index hospital admission, stratified by principal diagnosis

Principal diagnosis	CTO group with diagnosis *n* (%)	OR (95% CI)	OR_adj_ (95% CI)^a^	Sig.
Non-affective psychosis (incl. schizophrenia)	4108 (74.6)	0.72 (0.64–0.82)	0.67 (0.59–0.77)	**<0.0001**
Non-affective psychosis (excl. drug-induced psychosis)	3858 (70.1)	0.72 (0.64–0.80)	0.68 (0.60–0.77)	**<0.0001**
Mood disorders	742 (13.5)	0.99 (0.85–1.16)	1.04 (0.88–1.23)	0.622
All other diagnoses	628 (11.4)	1.51 (1.16–1.97)	1.55 (1.17–2.04)	**0.002**

CTO, Community Treatment Order; OR, odds ratio; OR_adj_, adjusted odds ratio; Sig., significance; incl., including; excl., excluding.

a. Adjusted for all variables in Table 2, except for non-affective psychosis (incl. schizophrenia).

Bold results indicate statistical significance (*p* < 0.05).

Table 4 shows the sociodemographic and health service use characteristics associated with median bed-days in the 12 months after the index psychiatric admission. People had significantly fewer median bed-days if they were aged 40 years or younger (*p* < 0.001) or were born outside of Australia, New Zealand or north-west Europe (*p* = 0.0001). People had a significantly higher median number of bed-days if they were living in a metropolitan area (*p* = 0.003), had any psychiatric and non-psychiatric admissions in the 12 months prior to the index psychiatric admission (*p* < 0.0001), greater than the median number of community mental health service contacts in the previous 12 months (*p* < 0.0001) or discharged on to a CTO (*p* < 0.0001). Stratification of people discharged on to a CTO by principal diagnosis revealed no significant differences in adjusted median bed-days in the 12 months after index psychiatric admission except for all other diagnoses (Table 5 and Fig. 1(b)). Sensitivity analyses excluding people with drug-induced psychosis revealed no significant difference, nor did sensitivity analyses restricted to psychiatric-specific hospital admissions in the following 12 months (Supplementary file 3).

**Table 4:** Sociodemographic and health service use characteristics associated with hospital bed-days over 12 months of follow-up, after the index hospitalisation

Characteristic	Unadjusted				Adjusted^a^			
	log *β* (95% CI)	s.e.	*t*	Sig.	log *β* (95% CI)	s.e.	*t*	Sig.
Age: ≤40 years old	−0.07 (−0.10, −0.03)	0.02	−3.47	**<0.001**	−0.07 (−0.11, −0.03)	0.02	−3.60	**<0.001**
Relationship status: Unpartnered/never married	0.08 (0.04, 0.11)	0.02	4.09	**<0.0001**	0.02 (−0.02, 0.06)	0.02	1.18	0.237
Sex: Male	−0.00 (−0.04, 0.03)	0.02	−0.19	0.852	0.01 (−0.02, 0.05)	0.02	0.75	0.456
Rurality of residence: Metropolitan area	0.06 (0.03, 0.10)	0.02	3.36	**<0.001**	0.06 (0.02, 0.10)	0.02	2.94	**0.003**
Country of birth: Outside of Australia, New Zealand and north-west Europe^b^	−0.12 (−0.17, −0.07)	0.02	−4.98	**<0.0001**	−0.11 (−0.16, −0.05)	0.03	−3.80	**0.0001**
Preferred language: Other than English	−0.14 (−0.21, −0.07)	0.04	−3.69	**<0.001**	−0.08 (−0.16, 0.00)	0.04	−1.90	0.057
Principal diagnosis: Non-affective psychosis (incl. schizophrenia)	0.13 (0.09, 0.16)	0.02	6.73	**<0.0001**	0.00 (−0.04, 0.04)	0.02	0.12	0.906
Any psychiatric admissions in previous 12 months^c^	0.45 (0.41, 0.49)	0.02	21.35	**<0.0001**	0.49 (0.44, 0.54)	0.02	20.22	**<0.0001**
Any non-psychiatric admissions in previous 12 months^c^	0.07 (0.04, 0.11)	0.02	3.79	**0.0001**	0.29 (0.24, 0.33)	0.02	13.14	**<0.0001**
Any community mental health appointments in previous 12 months, split by median	0.38 (0.35, 0.42)	0.02	20.46	**<0.0001**	0.22 (0.18, 0.26)	0.02	10.40	**<0.0001**
Discharged on to a CTO after index hospital admission	0.25 (0.21, 0.29)	0.02	12.71	**<0.0001**	0.12 (0.08, 0.17)	0.02	5.57	**<0.0001**

CTO, Community Treatment Order; *t*, ratio of the estimated coefficient to the s.e; Sig., significance; incl., including.

a. Adjusted for all variables in this table.

b. North-west Europe includes: UK, Channel Islands and Isle of Man (including England, Isle of Man, Northern Ireland, Scotland, Wales, Guernsey and Jersey), Ireland, western Europe (including Austria, Belgium, France, Germany, Liechtenstein, Luxembourg, Monaco, The Netherlands and Switzerland), northern Europe (including Denmark, Faroe Islands, Finland, Greenland, Iceland and Aland Islands).

c. Excluding the index admission.

Bold results indicate statistical significance (*p* < 0.05).

**Table 5:** Bed-days over 12 months of follow-up for people discharged on to a CTO after index hospital admission, stratified by principal diagnosis

Principal diagnosis	Unadjusted				Adjusted^a^			
	log *β* (95% CI)	s.e.	*t*	Sig.	log *β* (95% CI)	s.e.	*t*	Sig.
Non-affective psychosis (incl. schizophrenia)	−0.01 (−0.09, 0.07)	0.04	−0.34	0.735	−0.07 (−0.15, 0.01)	0.04	−1.62	0.104
Non-affective psychosis (excl. drug-induced psychosis)	−0.00 (−0.08, 0.07)	0.04	−0.06	0.951	−0.04 (−0.12, 0.04)	0.04	−0.95	0.341
Mood disorders	−0.07 (−0.17, 0.04)	0.05	−1.26	0.206	−0.02 (−0.12, 0.08)	0.05	−0.37	0.709
All other diagnoses	0.08 (−0.03, 0.19)	0.06	1.44	0.150	0.12 (0.01, 0.23)	0.06	2.20	**0.028**

CTO, Community Treatment Order; *t*, ratio of the estimated coefficient to the s.e; Sig., significance; incl., including; excl., excluding.

a. Adjusted for all variables in Table 4, except for non-affective psychosis (incl. schizophrenia).

Bold results indicate statistical significance (*p* < 0.05).

Supplementary file 4 shows the median number of community mental health appointments in the 12 months after the index psychiatric admission by principal diagnosis. Individuals with non-affective psychosis including schizophrenia and drug-induced psychosis had a median of 52 appointments (IQR 23–87, *p* < 0.0001). Individuals with non-affective psychosis excluding drug-induced psychosis had a median of 56 appointments (IQR 29–91, *p* < 0.0001). Individuals with mood disorders had a median of 19 appointments (IQR 6–50, *p* < 0.0001), and all other diagnoses had a median of 11 appointments (IQR 5–27, *p* < 0.0001). Within each diagnosis, people on a CTO had significantly more appointments than those receiving voluntary care.

## Discussion

This study expands on previous findings from Queensland^11^ by extending our methods to NSW – a more populous jurisdiction with levels of CTO use more representative of international rates. It is noteworthy that the majority of people discharged on to CTOs in NSW had a principal diagnosis of non-affective psychosis, the subgroup in which CTOs appeared to confer the greatest reduction in hospital readmissions. This pattern suggests that, at least diagnostically, CTOs are being applied to the population most likely to derive measurable benefit, even if the overall effectiveness remains limited. As in Queensland, we found that individuals discharged on to a CTO in NSW were no more likely to be readmitted within 12 months than those discharged into voluntary care. However, people with a principal diagnosis of non-affective psychosis (including schizophrenia and drug-induced psychosis) had significantly lower odds of hospitalisation after being discharged on to a CTO. This was over and above the fact that, irrespective of being on a CTO, people with this diagnosis were less likely to be admitted. Unlike the Queensland study, we found no significant difference in median bed-days for this group compared to those receiving voluntary care. While we did identify that being discharged on to a CTO was associated with significantly greater bed-days in the following 12 months across the full NSW sample, readmissions and bed-days represent only one dimension of CTO effectiveness. From the perspective of individuals experiencing CTO placement, other outcomes such as perceived autonomy, therapeutic alliance and quality of life may represent more meaningful indicators of CTO effectiveness. These findings contribute important insights to the growing evidence base advocating for more targeted use of CTOs, particularly in light of ongoing ethical and human rights concerns surrounding their widespread and variable application.

As is consistently shown in the literature,^6,9,22^ we found that people born outside of Australia, New Zealand or north-west Europe were more likely to be discharged on to a CTO following their psychiatric admission. Despite representing a significant proportion of the Australian population (25.7% of Australians were born in India, China, Philippines, Vietnam, South Africa, Nepal, Malaysia or Sri Lanka^23^), these individuals often face complex barriers to accessing community-based mental healthcare, including language difficulties, stigmatisation, racism and a lack of culturally appropriate services.^24^ While some researchers suggest that the high use of CTOs in the population is reflective of clinical need, their conclusions are based on unadjusted analyses using an ambiguous categorisation of CALD status.^25^ Instead, it appears more likely that the high use reflects attempts to compensate for systemic gaps in culturally safe and responsive care.^26,27^ However, this reliance on coercive treatment pathways risks compounding experiences of stigma, discrimination and disempowerment. While frameworks exist to support culturally inclusive service delivery,^28,29^ fundamental challenges remain, particularly in the collection and use of data that accurately reflect the cultural diversity of mental health service users.^30^ Addressing these disparities requires greater investment in culturally responsive care, including improved access to interpreters, voluntary community-based services and systemic reforms that promote equitable, culturally respectful treatment pathways.^31,32^ It also necessitates tackling the broader social determinants of health that disproportionately affect people from culturally diverse backgrounds.^31^

One possible reason for our finding that people with non-affective psychosis had lower odds of subsequent admissions, and especially those discharged on to a CTO, was that they had much greater follow-up with community mental health services in the following 12 months (Supplementary file 3). This is one of the main purposes of CTOs: to enforce adherence to treatment and medications in the community setting and limit the need for hospital readmission. However, CTOs can also function as an administrative mechanism that ensures timely community follow-up, particularly for individuals who services find difficult to work with.^33^ It may be that services lack the skills or resources to engage people experiencing non-affective psychosis, and must resort to using CTOs. Given the ongoing uncertainty about the effectiveness of CTOs – particularly regarding who benefits, under what conditions and through which mechanisms – there is a clear need to critically examine whether the potential benefits of CTOs are a function of coercion or simply reflect improved access to care that should be available voluntarily. If CTOs are being used to compensate for systemic service gaps,^34^ then greater investment in voluntary community-based care is necessary to offer a more ethical and sustainable alternative.

We identified approximately 5500 people who were discharged on to a CTO in NSW. While this is consistent with other NSW-wide figures,^35^ it is drastically lower than the number of people discharged on to a CTO in Queensland over the same period (*n* = 10 872).^11^ It is noteworthy that the NSW Mental Health Act 2007 stipulates that a CTO can be made for an affected person if there is no less restrictive care appropriate and available; a declared mental health facility has appropriate treatment for the individual and is able to implement it; and if the affected person has a history of refusing to accept appropriate care.^2^ The third clause is unique to NSW and may partly explain the significantly lower number of people compared to Queensland. Nonetheless, these disparities highlight the persistent and wide-ranging variability in CTO use across Australian jurisdictions, raising questions about the consistency and equity of placement and underscoring the need for evidence-informed, judicious use. Our findings suggest that, while CTOs may reduce hospital admissions for individuals with non-affective psychosis, this benefit does not extend to other diagnostic groups. The potential benefits for some must be carefully weighed against the coercive nature of CTOs and the ethical imperative to minimise restrictive practices, reserving their use for situations where less restrictive alternatives have been exhausted and a clear therapeutic benefit is anticipated.

### Limitations

This study has several limitations related to its design and data sources. First, we were unable to adjust for important individual-level variables such as SMI severity, forensic history, social disability or perceived risk of the individual to themselves or the community, all of which may influence treatment pathways and outcomes. Second, ethnicity was inferred using proxy indicators like country of birth and preferred language, which may not accurately reflect cultural identity, particularly for second- or third-generation individuals who still identify as culturally diverse. Due to small sample sizes, we also had to aggregate countries of origin into broader regional categories, which may have masked meaningful differences. For example, individuals from the UK and Ireland were grouped under north-west Europe, and our dataset did not distinguish between Māori and non-Māori individuals in New Zealand, despite known disparities in mental health outcomes. Because of data sovereignty concerns, we did not report individuals’ First Nations status, but First Nations people were included in the dataset. However, in contrast to involuntary admission, previous studies have generally shown that this population is not over-represented in the numbers of CTOs (though this may be a function of poor data quality rather than reality).^6,9^

Third, determining the legal status of individuals on CTOs also presented challenges. The MH-AMB data included expired legal status codes, which were excluded to avoid misclassification. However, it is unclear whether these codes were used in error or reflected actual CTO placements. As a result, some relevant cases may have been inadvertently omitted. Fourth, inclusion in the CTO group was based on an individuals’ first discharge from a psychiatric hospital admission on to a CTO during the study period, irrespective of whether this was their first ever or one of many previous discharges on to CTOs. Thus, the CTO group likely comprises both incident (new) and prevalent (existing) cases where the latter, by way of being admitted to hospital as a psychiatric in-patient, are indicating the ineffectiveness of their prior CTO placements. The high number of community mental health contacts in the prior 12 months amongst the CTO group also suggests prior CTO placement. However, this was adjusted for in regression models thereby limiting its effect on the findings.

Fifth, while we adjusted for several critical covariates, individuals discharged on to CTOs were more likely to be diagnosed with non-affective psychosis and had higher rates of prior admissions and community mental health appointments, suggesting they may have been more severely unwell than controls. Additionally, comparisons were made against standard community care; outcomes might differ if CTOs were compared to more intensive interventions such as assertive outreach.

Sixth, we did not undertake causal inference analyses. Whilst these can strengthen causal interpretation in observational studies, several key assumptions required for valid counterfactual estimation could not be satisfied. Important aspects of CTO placement and subsequent service use (e.g., illness severity, patient insight, social support, treatment adherence and clinical risk assessment) were not available in the administrative data, making residual confounding unavoidable.

Seventh, we were also unable to examine the duration of CTOs relative to voluntary treatment. A previous NSW study that linked Mental Health Review Tribunal and administrative health data, found that the effects of CTOs in comparison to voluntary controls were limited to when they were in force, particularly in the case of orders that lasted more than 2 years.^35^ Finally, our outcome measures were limited to hospital admissions and bed-days. While these are important indicators of service use, they do not capture broader clinical or psychosocial outcomes that may be more meaningful to individuals with SMI. The interpretation of reduced admissions as a positive outcome is also debated, which is why we included bed-days as a potentially more robust measure.

In all, this study suggests the need for more targeted and equitable use of CTOs in NSW, and greater consideration of whether CTOs are being used to compensate for systemic service gaps. While CTOs may reduce hospital admissions for individuals with non-affective psychosis, discharge on to a CTO had limited impact on hospital re-admission and length of stay for other diagnostic groups. These findings underscore the importance of refining CTO use to ensure it is clinically justified, culturally sensitive and ethically sound. Alternative, less coercive models of care that prioritise outcomes aligned with the goals of people with lived and living experience of SMI need to be explored.

## Supporting information

Bull et al. supplementary material 1 Bull et al. supplementary material

Bull et al. supplementary material 2 Bull et al. supplementary material

Bull et al. supplementary material 3 Bull et al. supplementary material

Bull et al. supplementary material 4 Bull et al. supplementary material

## Data Availability

Data availability is not applicable to this article as no new data were created or analysed in this study.

## Supplementary material

The supplementary material is available online at https://doi.org/10.1192/bjo.2026.10987
