# Supplementary material for: Factors influencing the outcomes of Community Treatment Orders: state-wide study using linked administrative health data from New South Wales, Australia
Source: BJPsych Open. 2026 Mar 10;12(2):e82. doi: 10.1192/bjo.2026.10987 (PMC13107322; doi:10.1192/bjo.2026.10987)
Supplement: Bull et al. supplementary material 1 — Bull et al. supplementary material [file S2056472426109879sup001.docx]

First (index) psychiatric admission on 01/02/2019

Accessed community mental health service on 13/03/2019

Discharged from psychiatric admission **voluntarily** on 30/02/2019

Health service use followed up until 29/02/2020

27 year old male

Control

1 January 2018

31 December 2022

27 year old male

Case

1 January 2018

31 December 2022

First (index) psychiatric admission on 01/01/2019

Discharged from psychiatric admission **on a CTO** on 01/02/2019

Health service use followed up until 31/01/2020

**Supplementary file 1:** Illustration of a case-control match
