# Supplementary material for: Factors influencing the outcomes of Community Treatment Orders: state-wide study using linked administrative health data from New South Wales, Australia
Source: BJPsych Open. 2026 Mar 10;12(2):e82. doi: 10.1192/bjo.2026.10987 (PMC13107322; doi:10.1192/bjo.2026.10987)
Supplement: Bull et al. supplementary material 2 — Bull et al. supplementary material [file S2056472426109879sup002.docx]

**Supplementary file 2:** Sociodemographic and health service use characteristics of people discharged from a psychiatric inpatient admission onto voluntary vs involuntary (CTO) care

| **Characteristic** | **Voluntary group**  9,761 (63.9%) | **CTO group**  5,506 (36.1%) |
| --- | --- | --- |
| Average age in years (SD) | 43.4±13.9 | 43.3±13.7 |
| Relationship status |  |  |
| Unpartnered/never married  Partnered/married | 4,847 (49.7%)  4,914 (50.3%) | 3,734 (67.8%)  1,772 (32.2%) |
| Sex^a^ |  |  |
| Male  Female | 5,904 (60.5%)  3,853 (39.5%) | 3,353 (60.9%)  2,151 (39.1%) |
| Rurality of residence |  |  |
| Remote or rural  Metropolitan | 4,307 (44.1%)  5,454 (55.9%) | 2,153 (39.1%)  3,353 (60.9%) |
| Country of birth |  |  |
| Australia, New Zealand and Northwest Europe^b^  Elsewhere | 8,159 (83.6%)  1,602 (16.4%) | 4,356 (79.1%)  1,150 (20.9%) |
| Preferred language |  |  |
| English  Other | 9,160 (93.8%)  601 (6.2%) | 5,082 (92.3%)  424 (7.7%) |
| Principal diagnosis |  |  |
| SUDs  Non-affective psychosis (incl. schizophrenia)  Non-affective psychosis (excl. drug-induced psychosis)  Mood disorders  Non-psychotic disorders (incl. anxiety)  Personality disorders  All other disorders | 749 (7.7%)  2,945 (30.2%)  2,247 (23.0%)  2,448 (25.1%)  1,843 (18.9%)  510 (5.2%)  1,258 (12.9%) | 99 (1.8%)  4,108 (74.6%)  3,858 (70.1%)  742 (13.5%)  204 (3.7%)  97 (1.8%)  228 (4.1%) |
| Median number of psychiatric admissions in previous 12-months (IQR) | 2 (1-3) | 2 (1-3) |
| Median number of non-psychiatric admissions in previous 12-months (IQR) | 2 (1-3) | 2 (1-3) |
| Median number of community mental health appointments in previous 12-months (IQR) | 7 (2-25) | 39 (14-82) |

^a^n=6 missing data (0.04%); ^b^Northwest Europe includes: United Kingdom, Channel Islands and Isle of Man (incl. England, Isle of Man, Northern Ireland, Scotland, Wales, Guernsey and Jersey), Ireland, Western Europe (incl. Austria, Belgium, France, Germany, Liechtenstein, Luxembourg, Monaco, Netherlands and Switzerland), Northern Europe (Denmark, Faroe Islands, Finland, Greenland, Iceland and Aland Islands); CTO = Community Treatment Order; SD = Standard deviation; LHD = Local Health District; SUDs = Substance Use Disorders; IQR = Interquartile range; NZ = New Zealand.
