# Supplementary material for: Factors influencing the outcomes of Community Treatment Orders: state-wide study using linked administrative health data from New South Wales, Australia
Source: BJPsych Open. 2026 Mar 10;12(2):e82. doi: 10.1192/bjo.2026.10987 (PMC13107322; doi:10.1192/bjo.2026.10987)
Supplement: Bull et al. supplementary material 3 — Bull et al. supplementary material [file S2056472426109879sup003.docx]

**Supplementary file 3:** Sensitivity analyses for psychiatric-specific hospitalisations and bed-days in the 12-months after the index hospitalisation

**Table 1:** Sociodemographic and health service use characteristics associated with psychiatric-specific hospital readmissions over 12-months of follow-up, after the index hospitalisation

| **Characteristic** | **Not admitted**  n=11,431 (74.9%)  ***n* (column %)** | **Admitted^a^**  n=3,836 (25.1%)  ***n* (column %)** | **OR (95%CI)** | **OR_adj_ (95%CI)^b^** | **Sig.** |
| --- | --- | --- | --- | --- | --- |
| Age: ≤40 years old | 5,166 (45.2%) | 1,902 (49.6%) | 1.19 (1.11-1.28) | 1.16 (1.07-1.26) | **<0.001** |
| Relationship status: Unpartnered/never married | 6,263 (54.8%) | 2,318 (60.4%) | 1.26 (1.17-1.36) | 1.04 (0.96-1.14) | 0.321 |
| Sex: Male | 6,946 (60.8%) | 2,311 (60.3%) | 0.98 (0.91-1.06) | 0.98 (0.90-1.06) | 0.584 |
| Rurality of residence: Metropolitan area | 4,981 (43.6%) | 1,479 (38.6%) | 1.23 (1.14-1.33) | 1.29 (1.19-1.40) | **<0.0001** |
| Country of birth: Outside of Australia, New Zealand and Northwest Europe^b^ | 2,182 (19.1%) | 570 (14.9%) | 0.74 (0.67-0.82) | 0.79 (0.70-0.89) | **<0.001** |
| Preferred language: Other than English | 846 (7.4%) | 179 (4.7%) | 0.61 (0.52-0.72) | 0.69 (0.57-0.83) | **0.0001** |
| Principal diagnosis: Non-affective psychosis (incl. schizophrenia) | 5,161 (45.1%) | 1,892 (49.3%) | 1.18 (1.10-1.27) | 0.87 (0.80-0.95) | **0.003** |
| Any psychiatric admissions in previous 12-months^c^ | 2,272 (19.9%) | 1,754 (45.7%) | 3.40 (3.14-3.67) | 3.66 (3.31-4.04) | **<0.0001** |
| Any non-psychiatric admissions in previous 12-months^c^ | 4,098 (35.8%) | 1,201 (31.3%) | 0.82 (0.75-0.88) | 1.58 (1.43-1.74) | **<0.0001** |
| Any community mental health appointments in previous 12-months, split by median | 5,140 (45.0%) | 2,459 (64.1%) | 2.19 (2.03-2.36) | 1.50 (1.37-1.63) | **<0.0001** |
| Discharged onto a CTO after index hospital admission | 3,776 (33.0%) | 1,730 (45.1%) | 1.66 (1.55-1.79) | 1.36 (1.24-1.49) | **<0.0001** |

^a^Adjusted for all variables in this Table; ^b^Northwest Europe includes: United Kingdom, Channel Islands and Isle of Man (incl. England, Isle of Man, Northern Ireland, Scotland, Wales, Guernsey and Jersey), Ireland, Western Europe (incl. Austria, Belgium, France, Germany, Liechtenstein, Luxembourg, Monaco, Netherlands and Switzerland), Northern Europe (Denmark, Faroe Islands, Finland, Greenland, Iceland and Aland Islands); ^c^Excluding the index admission; CTO = Community Treatment Order; CI = Confidence Interval; OR = Odds Ratio; OR_adj_ = Adjusted Odds Ratio.

**Table 2:** Odds of psychiatric-specific hospital readmissions over 12-months of follow-up for people placed on CTOs after index hospital admission, stratified by principal diagnosis

| **Principal diagnosis** | **CTO group with diagnosis**  ***n* (%)** | **OR (95%CI)** | **OR_adj_ (95%CI)^a^** | **Sig.** |
| --- | --- | --- | --- | --- |
| Non-affective psychosis (incl. schizophrenia) | 4,108 (74.6%) | 0.86 (0.76-0.98) | 0.77 (0.67-0.88) | **<0.001** |
| Non-affective psychosis (excl. drug-induced psychosis) | 3,858 (70.1%) | 0.85 (0.75-0.96) | 0.79 (0.69-0.90) | **<0.001** |
| Mood disorders | 742 (13.5%) | 0.92 (0.78-1.09) | 1.03 (0.86-1.23) | 0.767 |
| All other diagnoses | 628 (11.4%) | 1.39 (1.06-1.83) | 1.48 (1.12-1.97) | **0.007** |

^a^Adjusted for all variables in Table 1, except for non-affective psychosis (incl. schizophrenia); CTO = Community Treatment Order; OR = Odds Ratio; CI = Confidence Interval; OR_adj_ = Adjusted Odds Ratio; SUDs = Substance use disorders.


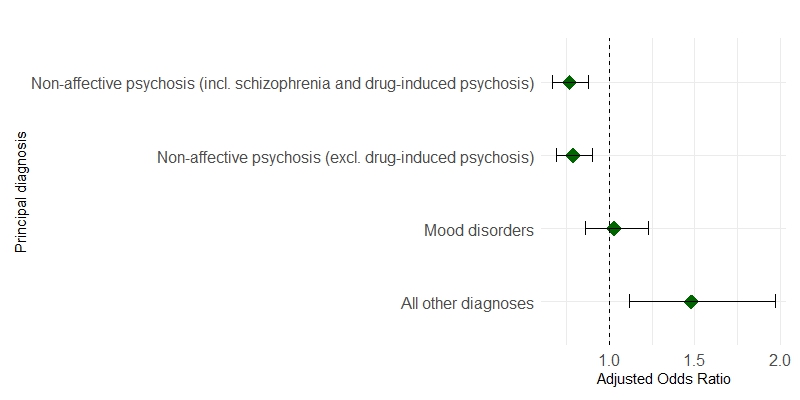


**Figure 1:** Adjusted odds of psychiatric-specific hospital admissions in the 12-months after index psychiatric admission for people discharged onto a CTO, stratified by principal diagnosis

**Table 3:** Sociodemographic and health service use characteristics associated with psychiatric-specific hospital bed-days over 12-months of follow-up, after the index hospitalisation

| **Characteristic** | **Unadjusted** | | | | **Adjusted^a^** | | | |
| --- | --- | --- | --- | --- | --- | --- | --- | --- |
|  | **log β (95%CI)** | **SE** | **t** | **Sig.** | **log β (95%CI)** | **SE** | **t** | **Sig.** |
| Age: ≤40 years old | 0.05  (0.01, 0.08) | 0.02 | 2.69 | **0.007** | 0.03  (-0.00, 0.07) | 0.02 | 1.73 | 0.083 |
| Relationship status: Unpartnered/never married | 0.15  (0.11, 0.18) | 0.02 | 8.13 | **<0.0001** | 0.03  (-0.00, 0.07) | 0.02 | 1.76 | 0.078 |
| Sex: Male | 0.03  (-0.01, 0.06) | 0.02 | 1.47 | 0.142 | 0.01  (-0.02, 0.05) | 0.02 | 0.73 | 0.465 |
| Rurality of residence: Metropolitan area | 0.06  (0.02, 0.09) | 0.02 | 3.24 | **0.001** | 0.05  (0.02, 0.09) | 0.02 | 2.80 | **0.005** |
| Country of birth: Outside of Australia, New Zealand and Northwest Europe^b^ | -0.07  (-0.12, -0.03) | 0.02 | -3.05 | **0.002** | -0.05  (-0.10, 0.00) | 0.03 | -1.80 | 0.072 |
| Preferred language: Other than English | -0.13  (-0.20, -0.06) | 0.04 | -3.51 | **<0.001** | -0.10  (-0.18, -0.02) | 0.04 | -2.51 | **0.012** |
| Principal diagnosis: Non-affective psychosis (incl. schizophrenia) | 0.24  (0.20, 0.27) | 0.02 | 13.32 | **<0.0001** | 0.08  (0.04, 0.12) | 0.02 | 3.84 | **<0.001** |
| Any psychiatric admissions in previous 12-months^c^ | 0.53  (0.50, 0.57) | 0.02 | 26.74 | **<0.0001** | 0.48  (0.44, 0.53) | 0.02 | 20.89 | **<0.0001** |
| Any non-psychiatric admissions in previous 12-months^c^ | -0.12  (-0.15, -0.08) | 0.02 | -6.18 | **<0.0001** | 0.11  (0.07, 0.15) | 0.02 | 5.27 | **<0.0001** |
| Any community mental health appointments in previous 12-months, split by median | 0.40  (0.37, 0.44) | 0.02 | 22.62 | **<0.0001** | 0.19  (0.15, 0.23) | 0.02 | 9.61 | **<0.0001** |
| Discharged onto a CTO after index hospital admission | 0.35  (0.31, 0.38) | 0.02 | 18.78 | **<0.0001** | 0.19  (0.14, 0.23) | 0.02 | 8.87 | **<0.0001** |

^a^Adjusted for all variables in this Table; ^b^Northwest Europe includes: United Kingdom, Channel Islands and Isle of Man (incl. England, Isle of Man, Northern Ireland, Scotland, Wales, Guernsey and Jersey), Ireland, Western Europe (incl. Austria, Belgium, France, Germany, Liechtenstein, Luxembourg, Monaco, Netherlands and Switzerland), Northern Europe (Denmark, Faroe Islands, Finland, Greenland, Iceland and Aland Islands); ^c^Excluding the index admission; CTO = Community Treatment Order; CI = Confidence Interval; SE = Standard Error; t = ratio of the estimated coefficient to the SE.

**Table 4:** Psychiatric-specific bed-days over 12-months of follow-up for people placed on CTOs after index hospital admission, stratified by principal diagnosis

| **Principal diagnosis** | **Unadjusted** | | | | **Adjusted^a^** | | | |
| --- | --- | --- | --- | --- | --- | --- | --- | --- |
|  | **log β (95%CI)** | **SE** | **t** | **Sig.** | **log β (95%CI)** | **SE** | **t** | **Sig.** |
| Non-affective psychosis (incl. schizophrenia) | 0.07  (-0.01, 0.14) | 0.04 | 1.63 | 0.103 | 0.00  (-0.08, 0.08) | 0.04 | 0.08 | 0.938 |
| Non-affective psychosis (excl. drug-induced psychosis) | 0.07  (-0.00, 0.15) | 0.04 | 1.87 | 0.062 | 0.03  (-0.04, 0.11) | 0.04 | 0.84 | 0.401 |
| Mood disorders | -0.06  (-0.17, 0.04) | 0.05 | -1.27 | 0.206 | -0.00  (-0.10, 0.10) | 0.05 | -0.04 | 0.969 |
| All other diagnoses | -0.04  (-0.15, 0.07) | 0.06 | -0.75 | 0.456 | 0.00  (-0.11, 0.11) | 0.06 | 0.03 | 0.978 |

^a^Adjusted for all variables in Table 3, except for non-affective psychosis (incl. schizophrenia); CTO = Community Treatment; CI = Confidence Interval; SE = Standard Error; t = ratio of the estimated coefficient to the SE.


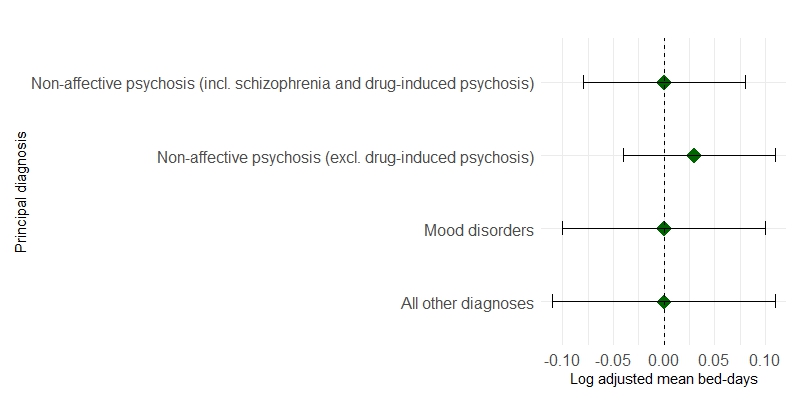


**Figure 2:** Log adjusted psychiatric-specific bed-days in the 12-months after index psychiatric admission for people discharged onto a CTO, stratified by principal diagnosis
