# Supplementary material for: Factors influencing the outcomes of Community Treatment Orders: state-wide study using linked administrative health data from New South Wales, Australia
Source: BJPsych Open. 2026 Mar 10;12(2):e82. doi: 10.1192/bjo.2026.10987 (PMC13107322; doi:10.1192/bjo.2026.10987)
Supplement: Bull et al. supplementary material 4 — Bull et al. supplementary material [file S2056472426109879sup004.docx]

**Supplementary file 4:** Median number of community mental health appointments over 12-months of follow-up, after the index hospitalisation, stratified by principal diagnosis

| **Principal diagnosis** | **Community mental health appointments over 12-months follow-up**  Median (IQR) | **Sig.** |
| --- | --- | --- |
| Non-affective psychosis (incl. schizophrenia) | 52 (23-87) | **<0.0001** |
| Discharged onto CTO (*n*=4,108)  Voluntary mental healthcare (*n*=2,945) | 66 (41-102)  27 (10-61) | **<0.0001** |
| Non-affective psychosis (excl. drug-induced psychosis) | 56 (29-91) | **<0.0001** |
| Discharged onto CTO (*n*=3,858) Voluntary mental healthcare (*n*=2,247) | 66 (42-102)  35 (13-68) | **<0.0001** |
| Mood disorders | 19 (6-50) | **<0.0001** |
| Discharged onto CTO (*n*=742)  Voluntary mental healthcare (*n*=2,448) | 54 (26-95)  14 (4-34) | **<0.0001** |
| All other diagnoses | 11 (5-27) | **<0.0001** |
| Discharged onto CTO (*n*=628)  Voluntary mental healthcare (*n*=4,360) | 33 (12-81)  10 (4-22) | **<0.0001** |

^a^Adjusted for all variables in this Table; CTO = Community Treatment Order; CI = Confidence Interval; OR = Odds Ratio; OR_adj_ = Adjusted Odds Ratio.
